# Baicalin attenuates cisplatin-induced cochlear hair cell damage by modulating the ROS-p38 MAPK signaling pathway

**DOI:** 10.3389/fcell.2026.1742764

**Published:** 2026-02-19

**Authors:** Qiongmin Zhang, Yating Wang, Chunhong Zhang, Sisi Li, Qian Yin, Chunchun Zhou, Chunguang Dong, Chuan Bu

**Affiliations:** 1 Department of Otorhinolaryngology, The First Affiliated Hospital of Wenzhou Medical University, Wenzhou, China; 2 Department of Otorhinolaryngology, The Affiliated Lianyungang Hospital of Xuzhou Medical University/The First People’s Hospital of Lianyungang, Lianyungang, China; 3 Department of Otorhinolaryngology, The First Affiliated Hospital of Kangda College of Nanjing Medical University/The First People’s Hospital of Lianyungang, Lianyungang, China

**Keywords:** apoptosis, baicalin, cisplatin, cochlear hair cell, ROS/P38 MAPK signaling

## Abstract

**Introduction:**

Cisplatin-induced ototoxicity remains a major clinical challenge in chemotherapy, with limited pharmacological strategies available to prevent auditory damage. In this study, we explored the protective potential of baicalin, a flavonoid compound, against cisplatin-triggered cochlear injury.

**Methods:**

*In vivo*, baicalin was administered to C57BL/6 mice prior to cisplatin treatment. Auditory function was assessed using auditory brainstem response (ABR) and distortion product otoacoustic emission measurements, and cochlear hair cell integrity was examined. *In vitro*, both House Ear Institute-Organ of Corti 1 (HEI-OC1) auditory cells and cochlear explants were used. Cell viability, apoptosis, mitochondrial reactive oxygen species (ROS) accumulation, and mitochondrial membrane potential (ΔΨm) were evaluated using MitoSOX Red, TMRM, and JC-1 fluorescence probes. The involvement of the p38 MAPK pathway was investigated using anisomycin (an activator) and SB203580 (an inhibitor) at the protein level.

**Results:**

*In vivo*, baicalin administration significantly mitigated cisplatin-induced hearing loss, as evidenced by improved ABR and distortion product otoacoustic emission thresholds and preserved cochlear outer hair cell structural and functional integrity. *In vitro*, baicalin pretreatment significantly improved cell viability and attenuated cisplatin-induced apoptosis. Mechanistically, baicalin markedly reduced mitochondrial ROS accumulation and maintained ΔΨm. Furthermore, baicalin pretreatment effectively inhibited p38 MAPK activation; this protective effect was reversed by anisomycin and mimicked by SB203580.

**Discussion:**

Collectively, these findings demonstrate that baicalin provides robust otoprotection against cisplatin-induced hearing loss. *In vitro* studies further indicate that this protective effect is associated with the attenuation of oxidative stress, maintenance of mitochondrial integrity, and inhibition of p38 MAPK–mediated apoptosis. Together, the results highlight baicalin as a promising candidate for therapeutic intervention against cisplatin-induced hearing loss.

## 1 Introduction

Drug-induced hearing loss (DIHL) is a common form of sensorineural hearing loss that severely impairs patients’ quality of life and imposes a substantial socioeconomic burden. Among ototoxic agents, cisplatin—a widely used chemotherapy drug—remains notorious for its irreversible damage to cochlear hair cells (HCs) and spiral ganglion neurons (Kros and Steyger, 2019), resulting in permanent hearing impairment in up to 40%–60% of treated patients (Rybak et al., 2019). The ototoxic effects of cisplatin are primarily driven by excessive reactive oxygen species (ROS) accumulation, mitochondrial dysfunction, and the activation of pro-apoptotic signaling pathways that converge on cochlear HCs (Gentilin et al., 2019; Sheth et al., 2017). Importantly, cisplatin-induced ROS can exert their cytotoxic effects by modulating the p38 mitogen-activated protein kinase (MAPK) signaling pathway, which acts as a stress-responsive cascade that amplifies oxidative stress and promotes HC degeneration by inducing inflammatory and apoptotic processes (Davoudi et al., 2022; Tabu et al., 2011). Increasing evidence indicates that several pharmacologic agents, including vitamin C, curcumin, lactate, mitoquinone, ginkgolide B, and N-acetylcysteine, exert protective effects against cisplatin-induced ototoxicity, primarily through antioxidative, anti-inflammatory, and anti-apoptotic pathways (Yu et al., 2020). Notably, in 2022 the U.S. Food and Drug Administration (FDA) approved sodium thiosulfate (Pedmark®) to reduce the risk of cisplatin-induced ototoxicity in pediatric patients with localized, non-metastatic solid tumors. However, its indication is limited to children, and no FDA-approved therapies currently exist for adults or for patients who have already developed cisplatin-related hearing loss. This underscores the urgent need for novel therapeutic agents that can effectively prevent or mitigate cisplatin ototoxicity across broader patient populations.

Compared with conventional synthetic compounds, natural extracts possess several advantages in preventing cisplatin-induced ototoxicity, including multi-target actions, superior antioxidant capacity, and minimal systemic toxicity. A growing body of evidence suggests that natural compounds possessing antioxidant properties, including astaxanthin, resveratrol and nobiletin, hold promise in attenuating cisplatin-induced ototoxicity (Nan et al., 2022; Song et al., 2024; Rybak et al., 2023; Wu et al., 2024). Baicalin, a flavonoid compound isolated from the roots of Scutellaria baicalensis Georgi, has been shown to have antioxidant and anti-inflammatory activity, suggesting its potential as protective agents (Hu et al., 2021). While baicalein is known for its strong bioactivity, baicalin, its glucuronide form, is more abundant in Scutellaria baicalensis extracts and may offer distinct pharmacokinetic advantages (Wang et al., 2024). Baicalin has attracted increasing attention in recent years for its potential therapeutic effects on diseases associated with oxidative stress and inflammation, which are becoming increasingly prevalent and pose a serious threat to human health (Szkudelski and Szkudelska, 2023; Ganguly et al., 2022; Kim et al., 2022). In auditory research, baicalin has demonstrated efficacy in attenuating gentamicin-triggered HC loss and noise-induced hearing impairment by modulating oxidative stress and inflammatory pathways (Zhang and Yu, 2019; Rodriguez et al., 2017; Castañeda et al., 2019). One study revealed that baicalin improves auditory brainstem response thresholds and reduces HC apoptosis in noise-exposed mice, likely by suppressing lipoxygenase (LOX) (Kang et al., 2010). However, whether baicalin confers protection against cisplatin-induced ototoxicity remains underexplored, and the precise mechanisms have yet to be clarified.

This study addresses these gaps by systematically evaluating baicalin’s protective effects against cisplatin-induced ototoxicity *in vitro* and *in vivo*. Then through experiments with HEI-OC1 auditory cells, we explored the possible mechanisms underlying the protective effects of baicalin on cochlear hair cells. We hypothesize that baicalin’s otoprotective effects may arise from its ability to suppress mitochondrial ROS accumulation and inhibit the activation of the ROS-dependent p38 MAPK signaling pathway.

## 2 Materials and methods

### 2.1 Animals and drug administration

The study utilized C57BL/6 mice acquired from the SPF Animal Facility of Wenzhou Medical University (Wenzhou, China). For the purpose of creating animal models that replicate cisplatin-associated hearing damage, 7–8 weeks male mice weighing 18–20 g were randomly assigned to experimental and control groups. Only male mice were used in this study to minimize variability associated with sex-specific hormonal fluctuations. Age-matched adult male C57BL/6 J mice (postnatal week 7–8, 19.2 ± 0.8 g) underwent stratified randomization using a computer-generated sequence (GraphPad Prism 9.0), allocating subjects to experimental or control cohorts under SPF conditions. Baicalin (purity≥95%, Sigma-Aldrich, United States, 21967-41-9) was dissolved in a vehicle consisting of 10% DMSO, 40% PEG400(Polyethylene Glycol 400), and 50% sterile saline. Based on previous *in vivo* studies demonstrating the protective efficacy and acceptable safety profile of baicalin, a dose of 30 mg/kg was selected in the present study to evaluate its otoprotective effects against cisplatin-induced ototoxicity (Wen et al., 2023; Chong et al., 2025). Mice in the experimental groups received daily intraperitoneal injections of cisplatin (3 mg/kg, injection, Hengri), while those in the control group were administered 0.9% sterile saline or baicalin (30 mg/kg) for 7 consecutive days. To evaluate the otoprotective potential of baicalin against cisplatin-hearing loss, mice were intraperitoneally injected with baicalin (30 mg/kg), followed by an intraperitoneal administration of cisplatin (3 mg/kg) 2 h later once daily for 7 days. The baicalin dosage in our experiment was selected based on a prior study assessing chinese medicine formulas against age-related hearing loss (Shi et al., 2025). All animal procedures were conducted in compliance with protocols approved by the Animal Experimental Ethics Committee of The First Affiliated Hospital of Wenzhou Medical University (Approval No. WYYY-AEC-YS-2023-0479), adhering to the ethical principles outlined in the Guidelines for the Welfare and Ethical Review of Experimental Animals (2018).

### 2.2 Postnatal murine cochlear organotypic culture

C57BL/6 mouse pups at postnatal day 3 (P3) were humanely euthanized in compliance with institutional animal care protocols. Temporal bones were rapidly excised and immersed in ice-cold Hanks’ Balanced Salt Solution (Hyclone, United States) to maintain tissue viability. Under sterile stereomicroscopic guidance, the cochlear duct was isolated through sequential removal of the bony capsule, lateral wall structures, and Reissner’s membrane. Isolated cochlear specimens were then affixed to CellTaK-coated glass coverslips (Fisher Scientific, PA) and maintained in a culture medium consisting of DMEM/F12 (Invitrogen, United States) enriched with 10% FBS (Gibco, United States), neurotrophic supplements (1% N2 and 2% B27, Invitrogen), and 50 mg/mL ampicillin (Sigma, United States). Cultures were incubated under standard physiological conditions (37 °C, 5% CO_2_) for subsequent experimental procedures.

For cochlear explant experiments, baicalin was dissolved in DMSO and diluted in culture medium to the indicated final concentrations (final DMSO concentration <0.1%). After 24 h of stabilization in culture, explants were pretreated with baicalin (45 μM) for 2 h, followed by exposure to cisplatin (30 μM) for an additional 24 h in the continued presence or absence of baicalin. Control explants received an equivalent volume of vehicle.

### 2.3 ABR and DPOAE test

Auditory brainstem response (ABR) thresholds in anesthetized mice were measured using 4–32 kHz clicks/tone bursts. Mice received xylazine-ketamine anesthesia (8 mg/kg and 50 mg/kg, i. m.) and were maintained on a heating pad (37 °C). Subdermal electrodes were placed at the vertex (active), ipsilateral mastoid (reference), and contralateral mastoid (ground). Acoustic stimuli (4–32 kHz) were delivered via calibrated transducers in a soundproof chamber, with intensity reduced from 90 dB SPL in 5 dB steps until waveform disappearance. ABR recordings were acquired using TDT System III hardware (Tucker-Davis Technologies, United States).

Distortion product otoacoustic emissions (DPOAEs) were recorded with two primary tones (f1 and f2, f2/f1 = 1.2, f2 set 10 dB lower than f1) presented at intensities decreasing from 80 to 20 dB SPL in 5-dB steps. Responses at the 2f1–f2 frequency were analyzed, and thresholds were defined as the lowest intensity at which the emission exceeded the surrounding noise floor by at least two standard deviations. Measurements were obtained at 4, 8, 16, 24, and 32 kHz using an acoustic probe connected to the TDT system.

### 2.4 Hair cell counting and cochleogram plotting

Hair cell quantification was conducted following a modified method adapted from previous study (Zhang Q. et al., 2023). In summary, cochlear samples were immunostained with phalloidin to label HCs. After defining the regions of interest at low resolution (×20 magnification), at least two non-overlapping Z-stacks per turn were acquired using a ×40 objective. The lengths of selected cochlear segments were measured, and the total numbers of inner hair cells (IHCs) and outer hair cells (OHCs) in each segment were counted using ImageJ software. Hair cell loss was calculated for each 0.300 mm cochlear segment and normalized to cochlear length (0% = apex, 100% = base). Loss patterns of IHCs and OHCs were plotted along the tonotopic axis, and mean cochleograms were generated by averaging individual data using custom software.

### 2.5 HEI-OC1 cell culture and cell viability assessment

HEI-OC1 cells were obtained from the UCLA Technology Development Group (Los Angeles, CA, United States). In the investigation of cisplatin-induced ototoxicity, HEI-OC1 cells served as the principal model owing to their consistent metabolic vulnerability profile with mouse cochlear cells (Kalinec et al., 1999). HEI-OC1 auditory cells were cultured under permissive conditions (33 °C, 5% CO_2_) using DMEM (4.5 g/L glucose) supplemented with 10% FBS, 100 U/mL penicillin-streptomycin, and 50 μg/mL ampicillin (pH 7.4 adjusted with NaHCO_3_). Cells were passaged at 80% confluence using 0.25% trypsin-EDTA.

For cell viability assessment, HEI-OC1 cells were seeded into 96-well plates at a density of 5 × 10^3^cells per well and allowed to adhere overnight. Cells were pretreated with baicalin (30, 45, or 60 μM) for 2 h, followed by co-incubation with cisplatin (30 μM) for 24 h at 37 °C/5% CO_2_. Cell viability was assessed using the Cell Counting Kit-8 (CCK-8; Dojindo Laboratories, Japan, CK04) according to the manufacturer’s instructions. Briefly, 10 μL of CCK-8 reagent was added per well, plates were incubated at 37 °C for 2 h in the dark, and absorbance was read at 450 nm using a microplate reader (Bio-Rad, United States). In parallel, nuclear morphology was analyzed after 4% paraformaldehyde fixation and DAPI staining (1 μg/mL, 15 min) using fluorescence microscopy (Nikon Eclipse Ti2), with automated ImageJ quantification. All experiments used cells between passages 5-15.

#### 2.6 Immunostaining

Following *in vitro* culture, specimens (cochlear explants/HEI-OC1 cells) were fixed with 4% PFA (15 min), blocked with PBT-1 (PBS containing 5% donkey serum, 1% BSA, 0.1% Triton X-100, 0.02% sodium azide)for 60 min, and stained with DAPI (1:1000 in PBS with 0.1% Triton X-100/1% BSA, 60 min). For F-actin labeling, samples were co-stained with iFluor™ 488-phalloidin (0.1 μM; Shanghai Yesen, China, 40727ES75) and DAPI (0.5 μg/mL, Invitrogen, United States, D1306) in PBS for 30 min at room temperature in the dark. Mounted specimens were imaged using a Leica SP8 confocal microscope (Germany) with LAS X software.

#### 2.7 TUNEL

HC apoptosis was evaluated via TUNEL assay using the Click-iT Plus system (Invitrogen, United States, C10617) according to standardized protocols. Specimens were co-stained with DAPI nuclear marker (0.1 μg/mL) and iFluor™ 488-phalloidin (0.1 μM in PBS; Yesen) for 30 min at room temperature in the dark. Fluorescent imaging was conducted on a Leica SP8 confocal platform (Germany) equipped with LAS X software, employing 40× oil immersion optics for subcellular resolution.

### 2.8 Protein extraction and Western blot analysis

Protein extracts from cochlear explants and HEI-OC1 cells were prepared using ice-cold RIPA buffer (Protein Biotech, China, PB180001) with protease inhibitors (Sigma). Lysates were centrifuged (12,000 × g, 10 min, 4 °C), and supernatants quantified via BCA assay (Beyotime, Chian, P0012). Equal protein aliquots underwent SDS-PAGE (12% gel) and PVDF membrane transfer (Millipore). Membranes were blocked with 5% skim milk/TBST (1 h, RT), then incubated with primary antibodies (CST: cleaved Caspase-3 1:500, p38 1:1000, p-p38 1:2000; Abcam: Bax/Bcl-2 1:500; ZSGB-BIO: β-actin 1:1000) in 3% milk/TBST at 4 °C overnight. After washing, membranes were incubated with the corresponding secondary antibodies (1:5000, 1 h, RT). Protein bands were visualized using ECL reagents (Millipore) and quantified with ImageJ software, normalizing target proteins to β-actin. Signals were detected by ECL (Millipore) and quantified using ImageJ.

#### 2.9 ROS detection

Cochlear explants and HEI-OC1 cells were subjected to three washes with PBS pre-equilibrated to 37 °C. Following washing, samples were loaded with 5 μM MitoSOX (Thermo Fisher Scientific, United States, M36008) for 15 min at 37 °C in the dark. Following staining, the explants were fixed using 4% paraformaldehyde (PFA) for 15 min at room temperature. Fluorescence imaging was performed on a Leica SP8 confocal system equipped with LAS X acquisition software (Leica Microsystems).

### 2.10 Measurement of mitochondrial membrane potential (Δψm)

HEI-OC1 cells were seeded in 6-well plates (1 × 10^5^/mL) and cultured at 37 °C/5% CO_2_ for 24 h. Mitochondrial polarization status in cells was assessed using JC-1 (Beyotime Biotechnology, China, C2006) or tetramethylrhodamine methyl ester (TMRM, Invitrogen, United States, T668) probes. HEI-OC1 cells were incubated with 5 μg/mL JC-1 working solution at 37 °C for 20 min in the dark, followed by two washes with ice-cold JC-1 buffer (pH 7.4). Cells were then counterstained with DAPI (15 min, 25 °C) and immediately imaged by confocal microscopy. The ratio of red (aggregates) to green (monomers) fluorescence was quantified using ImageJ. For TMRM staining, HEI-OC1 cells were incubated with 15 nM TMRM in complete culture medium at 37 °C for 25 min in the dark. After incubation, cells were washed once with pre-warmed PBS and immediately subjected to confocal fluorescence imaging. Fluorescence intensity was quantified using ImageJ software.

### 2.11 Statistical analysis

Each experimental condition was assessed through a minimum of three independent repetitions. Results are presented as mean ± SEM. Statistical significance was determined using one-way ANOVA followed by Tukey’s *post hoc* test for multiple comparisons among groups in GraphPad Prism 10.0 and SPSS 20.0. A p-value <0.05 was considered statistically significant.

## 3 Results

### 3.1 Baicalin improves HC survival and hearing function in mice after cisplatin injury

**FIGURE 1:**
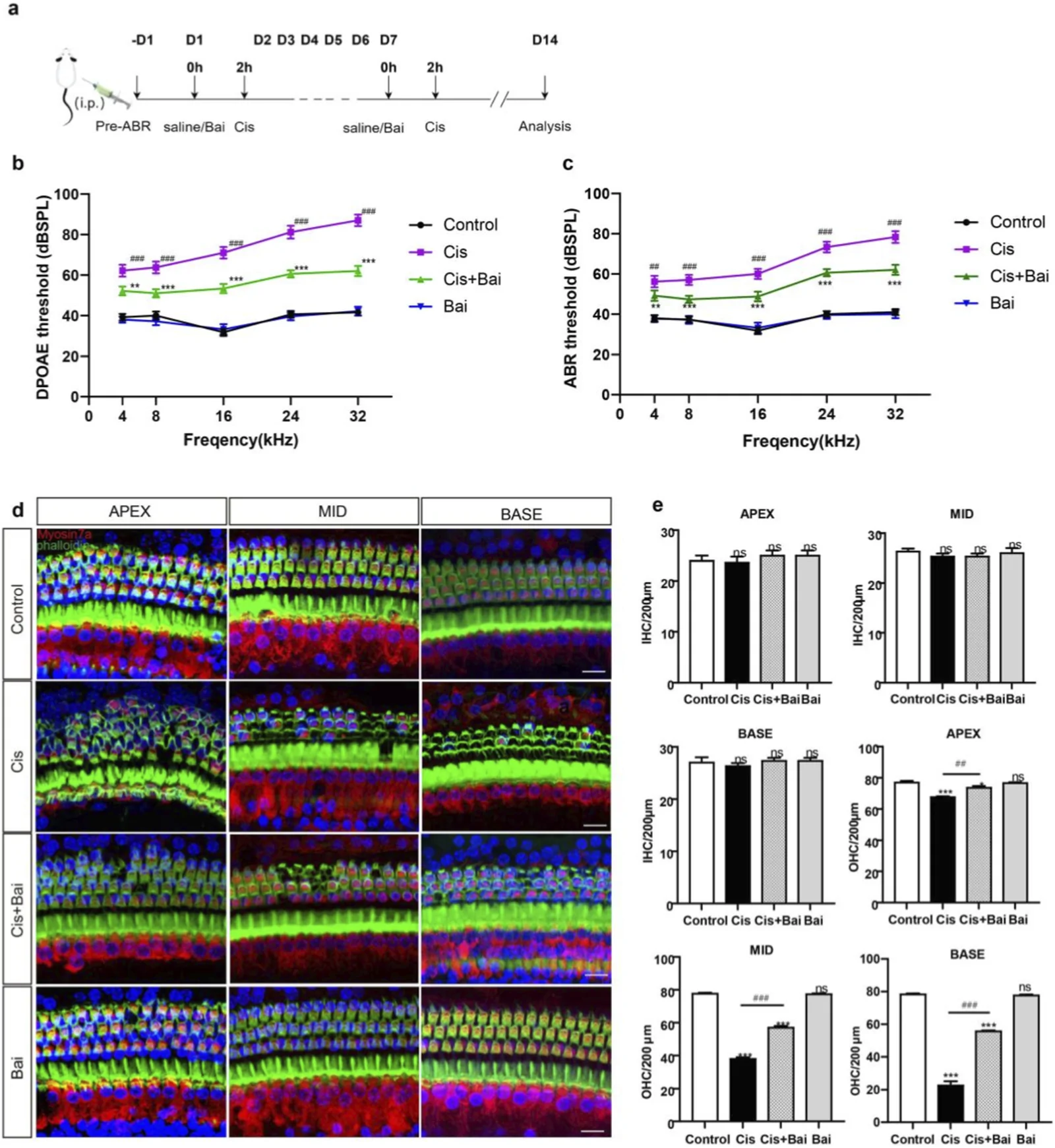
Baicalin improves HC survival and hearing function in mice after cisplatin injury. **(a)** The experimental workflow for **(b–e)**. **(b)** DPOAE thresholds at all frequencies from 4 to 32 were following intraperitoneal injection of saline, cisplatin, cisplatin plus baicalin (30 mg/kg) and baicalin only daily for 7 consecutive days. The Cis + Bai group exhibits reduced threshold shifts compared to Control. **(c)** ABR thresholds at all frequencies from 4 to 32 were measured 7 days after injection of control, cisplatin, cisplatin plus 30 mg/kg Baicalin, and 30 mg/kg Baicalin only. ABR thresholds demonstrate a similar trend, with the Cis group displaying elevated shifts across all frequencies, significantly mitigated in the Cis + Bai group. **(d)** Immunofluorescence staining with myosin7a (red) and phalloidin (green) in the apical, middle, and basal turns of cochleae from different groups. **(e)** The statistical analysis of HC counting (myosin7a, red) in the apical, middle, and basal turns of cochleae in each experimental groups. OHCs counts were markedly reduced in the Cis group at certain in all turns, with Bai showing protective effects in the Cis + Bai group. *P < 0.05, **P < 0.01,***p < 0.001,ns vs. the control group,^##^P < 0.01, ^###^P < 0.001, n = 6 cochlear explants derived from six individual P3 mice. Scale bar = 20 μm.

Previous study has shown baicalin alleviated gentamicin-triggered organ of Corti hair cell toxicity (Zhang and Yu, 2019), so we introduced baicalin in our study to determine whether it had protective effects on auditory cells against cisplatin. To assess baicalin’s protective efficacy *in vivo*, C57BL/6 mice were treated with baicalin at a dosage of 30 mg/kg body weight and subsequently injected with 3 mg/kg cisplatin 2 h later for 7 days, based on earlier studies (Wang et al., 2023; Guo et al., 2024) (Figure 1a). Six saline-treated animals without cisplatin or baicalin administration formed the control group. To evaluate the otoprotective efficacy of baicalin, auditory brainstem response (ABR) and distortion product otoacoustic emission (DPOAE) thresholds were systematically quantified across a range of frequencies (4, 8, 16, 24, 32 kHz) in murine subjects. As shown in Figure 1b, cisplatin administration resulted in a pronounced elevation of DPOAE thresholds at all tested frequencies compared with the control group (p < 0.001). Notably, the degree of impairment was more pronounced at higher frequencies (16–32 kHz), reflecting the basal turn vulnerability of cochlear hair cells to cisplatin injury. In contrast, animals co-treated with cisplatin and baicalin displayed significantly reduced DPOAE threshold shifts (p < 0.001 vs. cisplatin alone), indicating that baicalin effectively preserved OHC function. Similarly, ABR threshold shifts (Figure 1c) were markedly elevated following cisplatin exposure (p < 0.001 vs. control), but were substantially attenuated in the cisplatin + baicalin group (p < 0.001 vs. cisplatin), suggesting that baicalin protected against both hair cell dysfunction and auditory pathway impairment.

To corroborate these functional observations, we next examined the structural integrity of cochlear hair cells. Immunofluorescence staining for Myosin7a and phalloidin (Figure 1d) revealed that in control and baicalin-alone groups, the three rows of OHCs and one row of IHCs were well preserved throughout all cochlear turns. In contrast, cisplatin treatment induced extensive OHC loss, particularly in the middle and basal turns, while IHCs remained largely intact. Notably, baicalin co-treatment markedly reduced OHC loss and preserved the characteristic V-shaped stereocilia arrangement, especially in the basal turn. Quantitative analysis (Figure 1e) confirmed these findings: IHC counts showed no significant differences among groups at any cochlear turn, whereas OHC numbers were dramatically reduced by cisplatin (p < 0.001 vs. control). Co-administration of baicalin significantly preserved OHC survival at the apical, middle, and basal turns (p < 0.001 vs. cisplatin), whereas baicalin alone had no effect on hair cell counts.

### 3.2 Baicalin protects against cisplatin-induced ototoxicity in HEI-OC1 cells and cochlear hair cells

**FIGURE 2:**
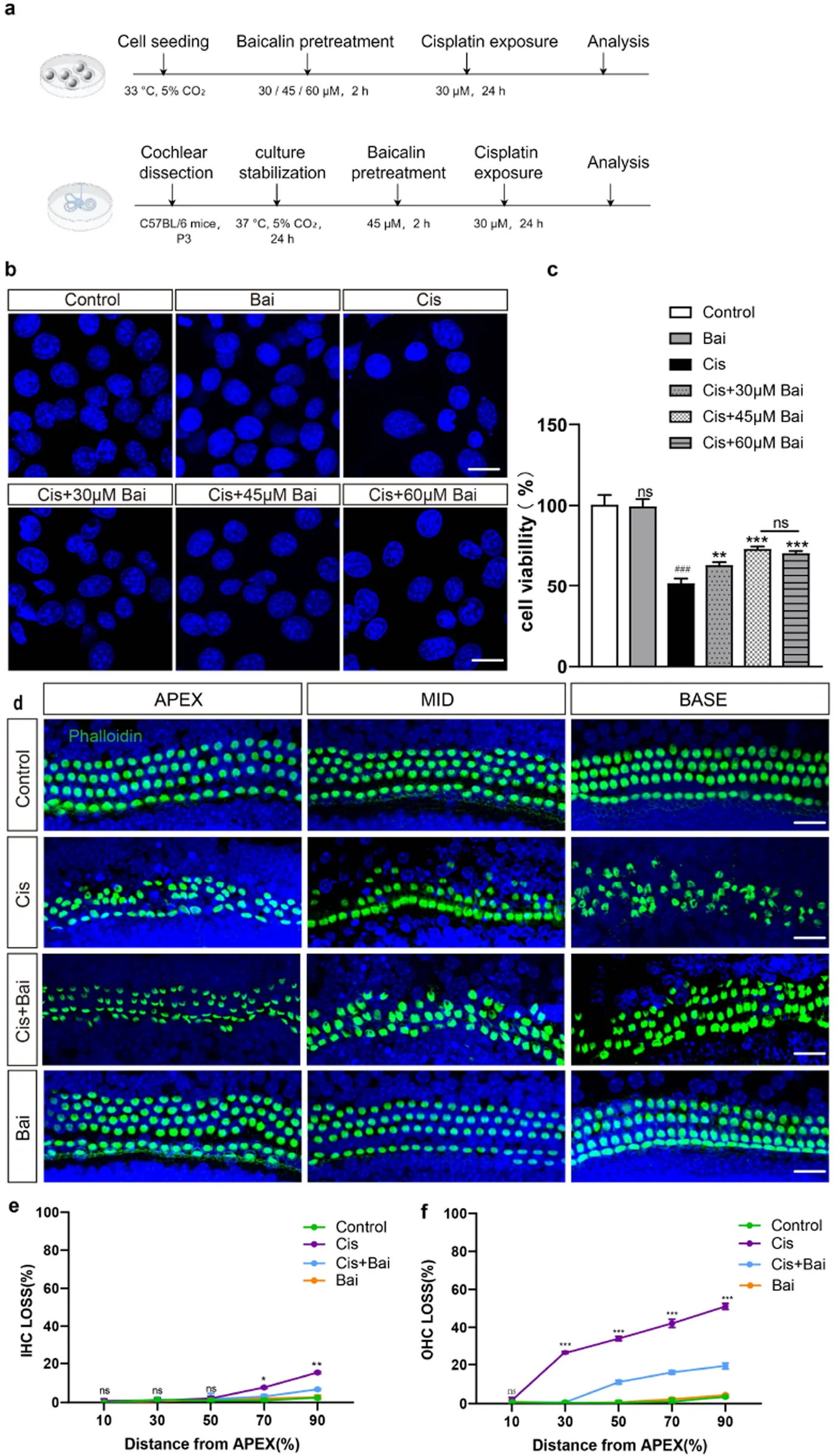
Baicalin protects against cisplatin-induced ototoxicity in HEI-OC1 cells and cochlear hair cells. **(a)** The experimental workflow for **(b–f)**. **(b)** Representative images of cell viability under different treatment conditions. Cisplatin (30 μM) treatment led to a marked decrease in cell viability relative to the untreated group. Co-treatment with various concentrations of baicalin (30, 45, 60 μM) partially restored cell viability in a dose-dependent manner. **(c)** Quantitative analysis of cell viability in different groups (control, Bai, Cis, Cis +30 μM Bai, Cis +45 μM Bai, and Cis +60 μM Bai). Data are presented as mean ± SD from three independent biological experiments, each performed in triplicate (n = 3). Statistical analysis was conducted using one-way ANOVA followed by Tukey’s *post hoc* test. ^###^p < 0.001 vs. control,**p < 0.01 vs. Cis,***p < 0.001 vs. Cis, ns = not significant. **(d)** Immunofluorescence images showing the organization of inner hair cells (IHCs) and outer hair cells (OHCs) across the cochlear apex, mid, and base regions. Phalloidin (green) staining shows the actin cytoskeleton of hair cells. Cisplatin-treated groups show HC loss, especially in the mid and base regions, while co-treatment with baicalin provided substantial protection against cisplatin-triggered hair cell injury. **(e,f)** The mean cochleogram showed HCs loss across different cochlear regions (apex to base) in control, Cis, Cis + Bai, and Bai groups. Significant HCs loss was observed in the Cis group, especially in the mid and base regions. Baicalin co-treatment reduced this loss. **p < 0.01, ***p < 0.001. For **(a,b)**, n = 6 independent biological replicates obtained from separate cell culture experiments. For **(c–e)**, n = 6 cochlear explants derived from six individual P3 mice. Scale bar = μm.

Encouraged by the *in vivo* evidence of baicalin’s protective role against cisplatin-induced hearing loss, we next sought to validate its protective effects at the cellular level. To this end, we employed HEI-OC1 auditory cells to investigate the underlying cellular responses and dose-dependent cytoprotective efficacy of baicalin against cisplatin-induced ototoxicity. HEI-OC1 auditory cells were pretreated with a concentration gradient of baicalin (30, 45, 60 μM) for 2 h, subsequently sustained in co-exposure to 30 μM cisplatin for an additional 24-h period (Figure 2a). Immunostaining findings revealed that HEI-OC1 subjected to cisplatin exhibited a gradual recovery in cell count reduction and nucleus deformation with the rising concentrations of baicalin (Figure 2b). Quantitative CCK-8 analysis indicated that dose-dependent viability of HEI-OC1 cells was significantly increased to 63.17% ± 2.06% following the administration of 30 μM baicalin compared with the control group, and it was further enhanced to 76.81% ± 2.20% at concentrations of 45 μM, 74.83% ± 2.09% at 60μM (Figure 2c). Based on dose-response optimization, a 24-h pretreatment with 45 μM baicalin was established as the standard protocol for subsequent HEI-OC1 cellular investigations.

To further validate these findings at the level of primary tissue cultures, we next investigated the protective role of baicalin in cisplatin-exposed cochlear hair cells (HCs). After baicalin pretreatment, cochleae were exposed to cisplatin with or without continued baicalin to evaluate differences in HC survival. Spatiotemporal patterns of hair cell degeneration were quantitatively mapped through immunocytochemistry and cochleogram analysis, with specific focus on quantifying IHCs and OHCs cell loss gradients across experimental cohorts. Consistent with the quantitative mapping in Figures 2d–f, cisplatin administration induced a tonotopic gradient of hair cell degeneration, with the loss of OHCs being more severe than that of IHCs, which aligns with prior research indicating that cisplatin disproportionately affects OHCs (Bu et al., 2022; Gibaja et al., 2022). Baicalin alone did not cause measurable HC loss. Importantly, co-treatment with baicalin significantly attenuated cisplatin-induced HC degeneration. The protective effect was evident across all cochlear regions but was particularly pronounced in the mid-basal and basal turns. Moreover, baicalin preserved IHCs more effectively in the basal segment, where cisplatin-induced loss was otherwise prominent. These findings indicate that baicalin confers significant protection against cisplatin ototoxicity, with preferential preservation of OHCs along the tonotopic axis and a stronger effect in the basal cochlea.

### 3.3 Baicalin attenuates cisplatin-induced apoptosis in HEI-OC1 cells and cochlear hair cells

**FIGURE 3:**
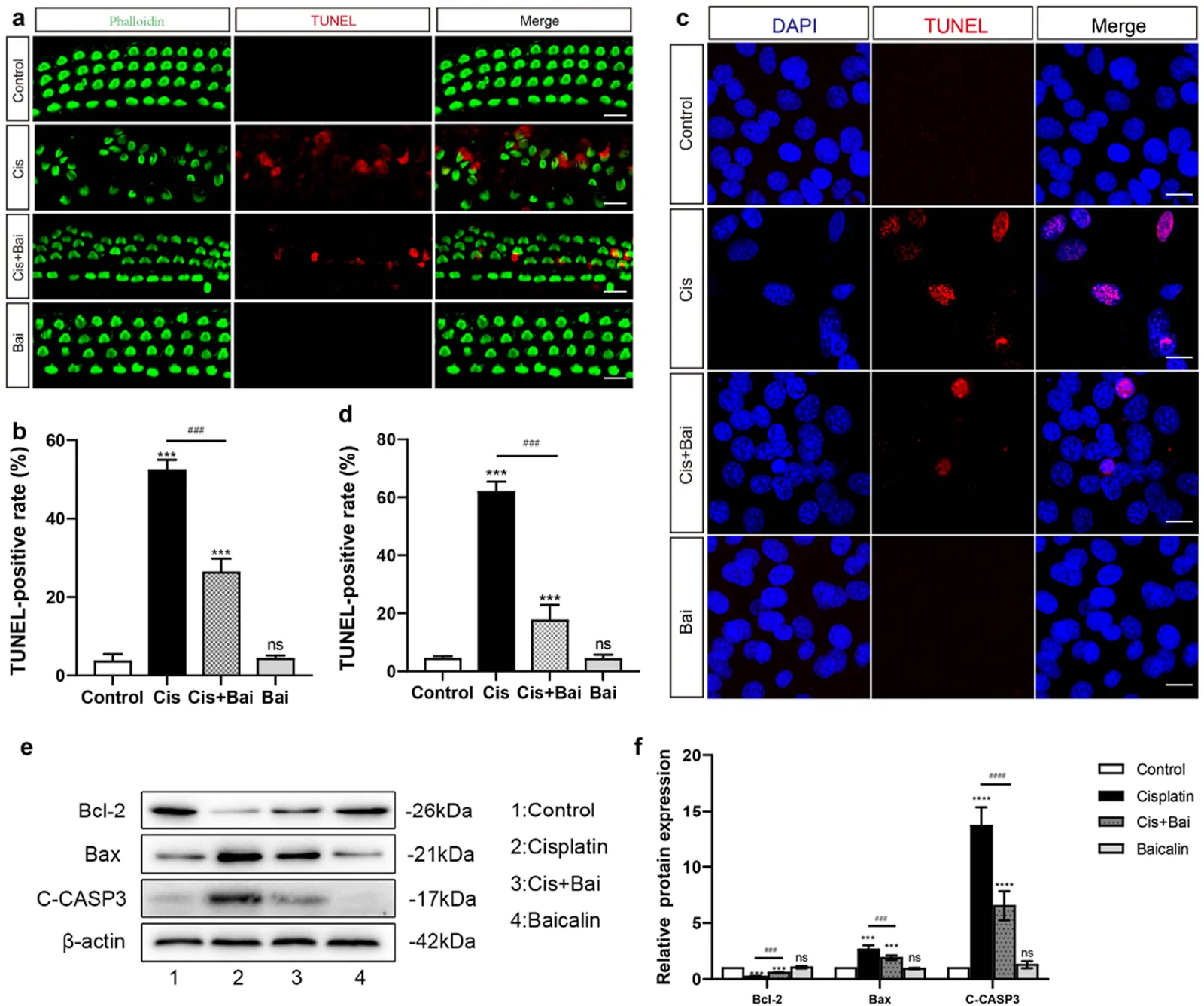
Baicalin protect cells from apoptosis in HEI-OC1 cell line and murine cochlear HCs after cisplatin injury *in vitro*. **(a,b)** Immunofluorescence images of cochlear hair cells showing p halloidin (green) and TUNEL (red) staining in the base regions. Cisplatin treatment resulted in increased TUNEL-positive apoptotic cells relative to the control group, while baicalin co-treatment notably decreased the quantity of apoptotic cells. **(c,d)** DAPI (blue) and TUNEL (red) staining of nuclei in HEI-OC1 cells, showing increased apoptotic cell death in the Cis group, while baicalin co-treatment significantly decreased TUNEL-positive cells. **(e,f)** Western blot analysis demonstrating quantitative alterations of Bcl-2, Bax, and cleaved C-CASP3 in different treatment groups. Cisplatin treatment decreased Bcl-2 level and elevated Bax and C-CASP3 levels. Baicalin co-treatment restored Bcl-2 levels and reduced Bax and C-CASP3 expression. Data are presented as mean ± SD (n = 3), ***p < 0.001,****p < 0.0001,ns vs. the control group, ^###^P < 0.001,^####^P < 0.0001, ns = not significant. For **(a,b)**, n = 6 cochlear explants derived from six individual P3 mice. For **(c,d)**, n = 6 independent biological replicates obtained from separate cell culture experiments. Scale bar = 20 μm.

To systematically evaluate the cytoprotective efficacy of baicalin on injury induced by cisplatin, the Terminal deoxynucleotidyl transferase-mediated dUTP nick-end labeling (TUNEL) assay was utilized to detect apoptotic cells, which provides a sensitive and reliable indication of DNA fragmentation as a hallmark of programmed cell death. As demonstrated in the representative fluorescence micrographs of Figures 3a,b, cisplatin-induced apoptotic damage in cochlear hair cells (HCs) was characterized by the co-localization of TUNEL-positive nuclei (red fluorescence) with preserved phalloidin immunostaining (green fluorescence). Notably, the cisplatin + baicalin group exhibited a markedly lower proportion of TUNEL-positive cells (27.35% ± 2.63%) in comparison to the cisplatin group (53.12% ± 2.18%), indicating that baicalin effectively attenuated cisplatin-triggered apoptotic cell death in the cochlea. Consistently, TUNEL results in HEI-OC1 cells further corroborated these findings, revealing that a high percentage of TUNEL-positive cells (61.02% ± 1.98%) was detected in the cisplatin group, whereas a significantly reduced percentage of TUNEL-positive cells (18.56% ± 2.23%) was observed in the cisplatin + baicalin group (Figures 3c,d). These *in vitro* results collectively highlight a reproducible anti-apoptotic effect of baicalin across different experimental models.

Moreover, mechanistic interrogation of apoptotic signaling pathways was performed through protein-level profiling of core apoptosis-regulatory genes. Compared to the cisplatin-only group, HEI-OC1 cells co-treated with cisplatin and baicalin for 24 h exhibited a significant downregulation of cleaved Caspase-3 and the pro-apoptotic protein Bax, accompanied by a pronounced upregulation of the anti-apoptotic protein Bcl-2 (p < 0.001) (Figures 3e,f). This molecular expression profile suggests that baicalin inhibits the intrinsic apoptotic cascade, thereby enhancing cell survival.

### 3.4 Baicalin prevents mitochondrial ROS accumulation in cochlear explants and HEI-OC1 cells after cisplatin injury

**FIGURE 4:**
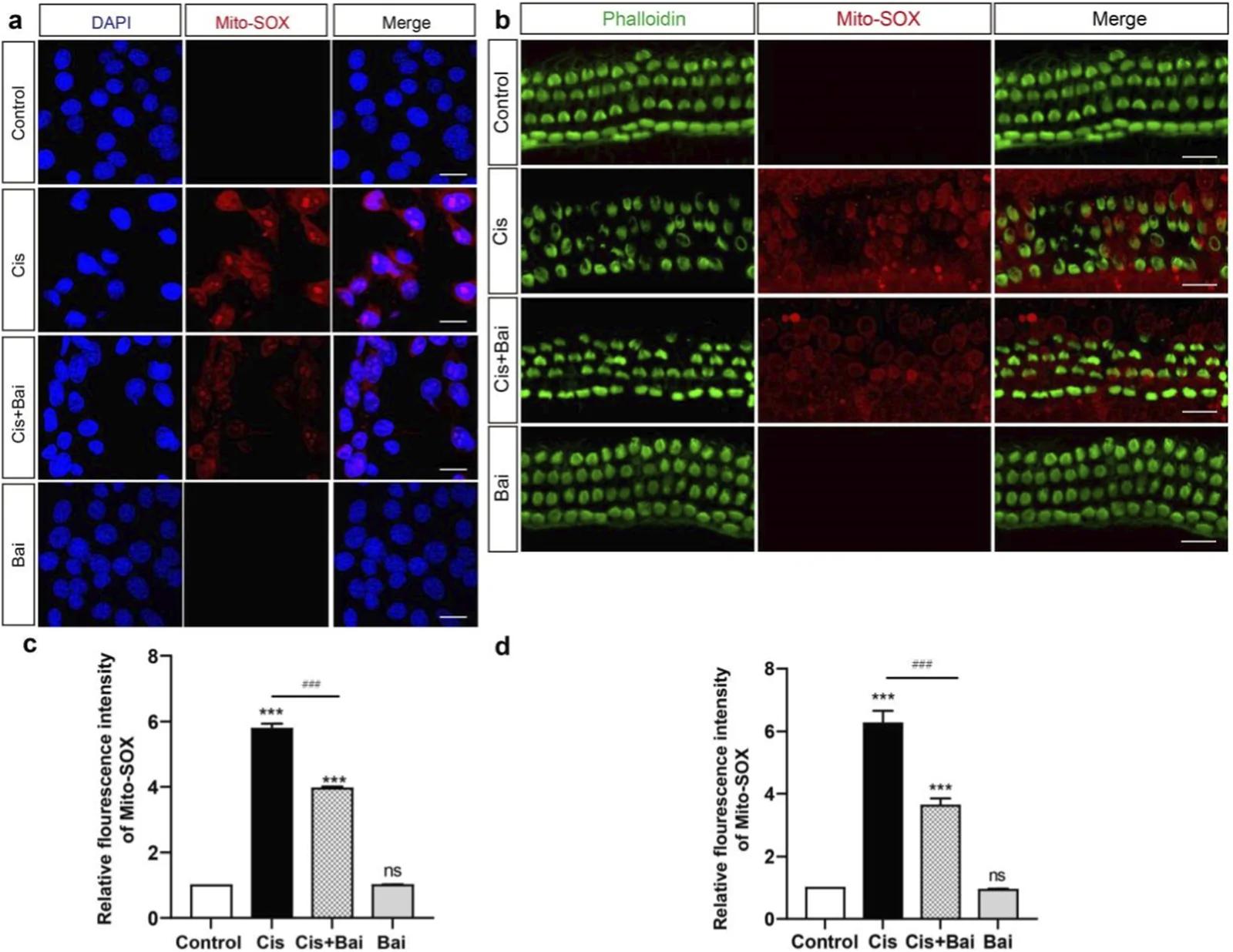
Baicalin prevents mitochondrial ROS accumulation in HEI-OC1 cells and cochlear explants after cisplatin damage. **(a–c)** Representative images of DAPI (blue) and Mito-SOX (red) in different treatment groups in HEI-OC1 cell line: Control, Cis, Cis + Bai, and Bai. The Control and Bai groups showed minimal Mito-SOX staining, indicating low ROS levels, while the Cis group showed significantly increased Mito-SOX intensity, indicating elevated ROS production. Treatment with Cis + Bai reduced ROS levels compared to Cis alone. **(b–d)** Representative images of fluorescence staining showing Phalloidin (green) and Mito-SOX (red)in the same treatment groups as a in cultrured cochlea. The relative Mito-SOX fluorescence intensity showed a significant elevation in mitochondrial ROS in the Cis group in contrast to Control, which was significantly diminished in Cis + Bai group. The Bai alone group exhibited no significant difference in contrast to Control. Fluorescence intensity was normalized to the control group (control = 1.0). ***p < 0.001, ns vs. the control group, ^###^P < 0.001, ns = not significant. For **(a–c)**, n = 6 independent biological replicates obtained from separate cell culture experiments. For **(b–d)**, n = 6 cochlear explants derived from six individual P3 mice. Scale bar = 20 μm.

ROS have been demonstrated to exhibit a strong correlation with the mechanism of HC injury caused by ototoxic medications (Thakur et al., 2024; Maniaci et al., 2024; Mirzaei et al., 2021), and excessive mitochondrial ROS production is widely recognized as a key initiator of oxidative stress–mediated cellular apoptosis. To investigate the association between baicalin and oxidative strain in auditory cells, Mito-SOX Red was employed to assess mitochondrial reactive oxygen species (ROS) buildup in HEI-OC1 cell line and cochlear explants following the different treatments. HEI-OC1 cells were incubated with baicalin before the co-treatment with cisplatin for 24 h, thereby enabling us to evaluate the potential preventive effect of baicalin on cisplatin-triggered oxidative injury. As shown in Figure 4a, the control group and the baicalin-only group exhibited virtually no MitoSox-Red–positive cells, suggesting that baicalin alone does not induce mitochondrial oxidative stress and that basal ROS levels in auditory cells remain low under physiological conditions. The relative fluorescence intensity of Mito-SOX Red exhibited a marked upregulation after 24 h of cisplatin exposure compared to the uninjured controls (p < 0.001), with this escalation being significantly attenuated by baicalin pretreatment (p < 0.01 vs. cisplatin alone) (Figures 4a,c), indicating that baicalin is able to effectively suppress cisplatin-induced mitochondrial ROS overproduction in auditory cells.

To provide molecular evidence for baicalin’s pharmacological inhibition of cisplatin-triggered ROS overproduction, MitoSOX Red mitochondrial superoxide staining with fluorescence intensity quantification was systematically performed in cultured hair cells (Figure 4b), thereby providing additional experimental evidence at the tissue level. The relative fluorescence intensity of MitoSOX Red in baicalin-cotreat group decreased substantially compared to the cisplatin-only group (p < 0.01) (Figures 4b,d), further confirming that baicalin markedly alleviates mitochondrial ROS accumulation within cochlear hair cells. Together, these findings strongly suggest that baicalin confers protection against cisplatin-induced oxidative stress both in auditory cell lines and in cochlear explants, thereby supporting its potential as a pharmacological agent for mitigating ototoxic injury.

### 3.5 Baicalin preserves mitochondrial membrane potential in cisplatin-damaged HEI-OC1 cells

**FIGURE 5:**
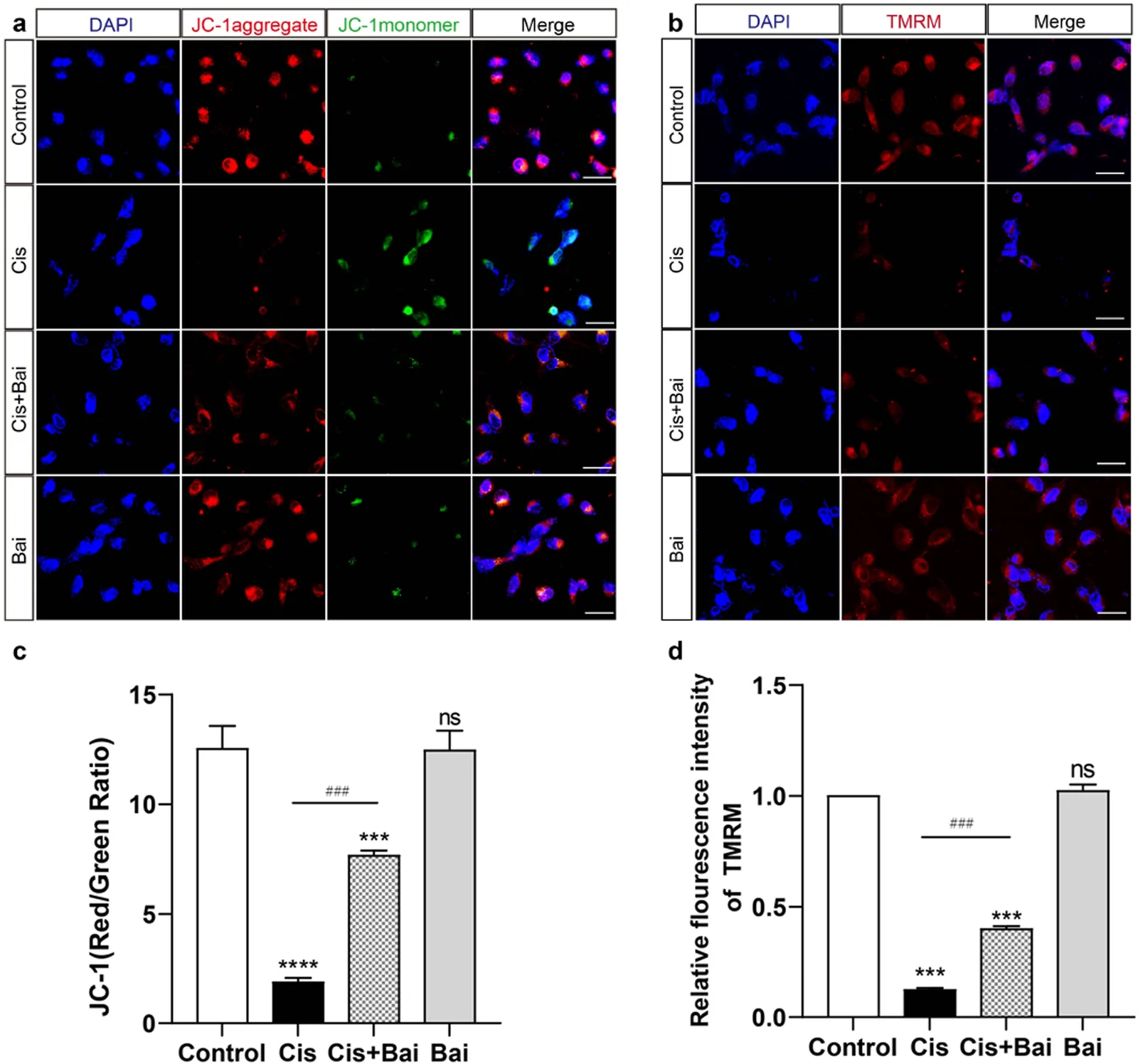
Baicalin preserves mitochondrial membrane potential in cisplatin-damaged HEI-OC1 cells. **(a–c)** JC-1 fluorescence micrographs and corresponding ratiometric analysis (aggregate/monomer fluorescence intensity) demonstrating Baicalin-mediated preservation of mitochondrial transmembrane potential in cisplatin-challenged HEI-OC1 auditory cells. The JC-1 fluorescence (red/green ratio) in Cis group significantly decreased, while co-treatment with Bai raised the JC-1 fluorescence (red/green ratio) in the Cis + Bai group. **(b–d)** Representative images of TMRM staining showed different mitochondria changes in the same treatment groups as a. The relative fluorescence intensity of TMRM was markedly decreased in the Cis group, reflecting a decrease in mitochondrial membrane potential, while the Cis + Bai group showed restored mitochondrial membrane potential to Cis alone. Fluorescence intensity was normalized to the control group (control = 1.0). ***p < 0.001, ****p < 0.0001,ns vs. the control group,^###^P < 0.001, ns = not significant. n = 6 independent biological replicates obtained from separate cell culture experiments. Scale bar = 20 μm.

Mitochondria are widely recognized as the principal generators of intracellular ROS and concurrently the organelle is most susceptible to ROS-triggered injury (Willems et al., 2015; Chakrabarty and Chandel, 2021), thereby serving as a central target for oxidative damage–mediated cellular dysfunction. To assess the primary indicator of mitochondrial dysfunction (Bazhin et al., 2020), we evaluated differences in mitochondrial membrane potential (ΔΨm) between baicalin-treated and untreated HEI-OC1 cells following cisplatin-induced damage using dual fluorescence detection with TMRM and JC-1 probes, both of which are established and complementary methods for monitoring mitochondrial polarization status. As depicted in Figures 5a,c, JC-1 probe exhibited characteristic mitochondrial aggregation with dominant red fluorescence in control HEI-OC1 cells, consistent with preserved transmembrane electrochemical polarity and intact ΔΨm. In contrast, cisplatin-exposed cells demonstrated a marked fluorescence shift towards green spectral dominance, reflecting JC-1 monomer predominance, which quantitatively confirmed ΔΨm collapse through fluorometric analysis (p < 0.001 vs. control). However, the JC-1 red/green fluorescence ratio notably increased in HEI-OC1 cells co-treated with baicalin (p < 0.001 vs. cisplatin alone), indicating partial restoration of membrane potential and suggesting that baicalin significantly counteracts cisplatin-induced mitochondrial depolarization.

Furthermore, TMRM staining showed a similar trend and served as an additional validation of ΔΨm changes. As shown in Figures 5b,d, the substantial reductions of fluorescence intensity of TMRM in the cisplatin-impaired cells also indicated the loss of ΔΨm (p < 0.001 vs. control), while baicalin treatment significantly restored TMRM fluorescence intensity (p < 0.01 vs. cisplatin alone). Collectively, these findings provide convergent evidence from two independent assays demonstrating that baicalin effectively preserves mitochondrial membrane potential and mitigates cisplatin-induced mitochondrial dysfunction in auditory cells.

### 3.6 Baicalin attenuates cisplatin-induced mitochondrial ROS and apoptosis by restoring p38 MAPK pathway

**FIGURE 6:**
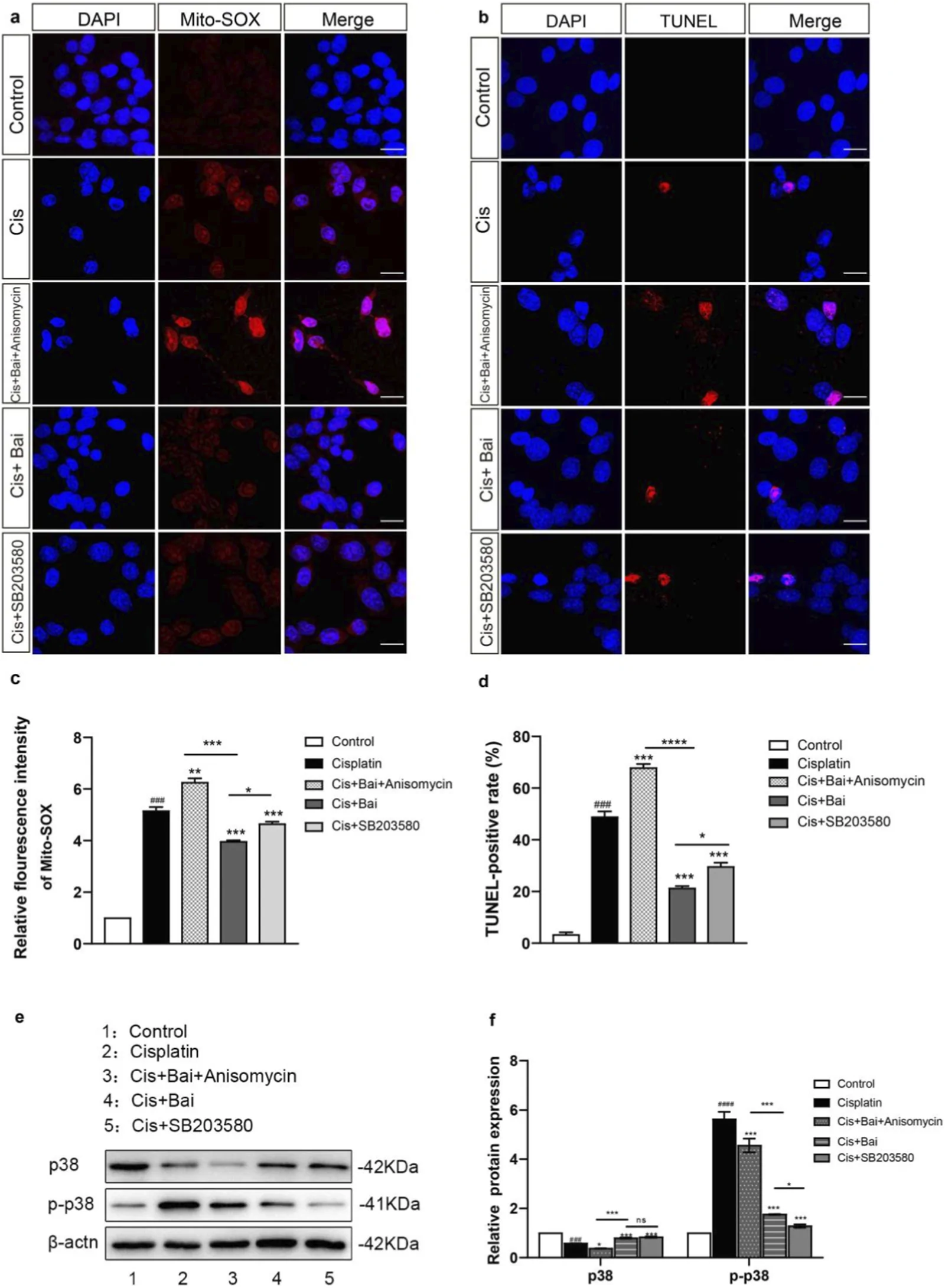
Baicalin attenuates cisplatin-induced ROS accumulation and apoptosis by restoring p38 MAPK pathway. **(a–c)** Representative fluorescence images of DAPI (blue) and MitoSOX™ Red (red) staining showing mitochondrial ROS levels in Control, Cis, Cis + Bai + Anisomycin, Cis + Bai, and Cis + SB203580 groups. Cisplatin exposure markedly increased MitoSOX fluorescence intensity compared with the Control group, indicating elevated ROS production. This increase was significantly reduced by Baicalin, partially reversed by the p38 agonist anisomycin, and mimicked by the p38 inhibitor SB203580. **(b–d)** Representative TUNEL staining (red) of apoptotic cells, with DAPI (blue) counterstaining of nuclei. Cisplatin treatment significantly increased the apoptosis rate compared with the Control group, whereas Baicalin co-treatment decreased TUNEL-positive cells. The anti-apoptotic effect of Baicalin was attenuated by anisomycin but reproduced by SB203580. **(e,f)** Relative protein expression of total p38 and phosphorylated p38 (p-p38), normalized to Control. Cisplatin treatment induced a significant upregulation of p-p38 and modest changes in p38 compared with Control. Baicalin markedly attenuated p-p38 expression, an effect diminished by anisomycin but mimicked by SB203580. β-actin served as a loading control. Data are presented as mean ± SEM. ^###^p < 0.001,^####^p < 0.0001 vs. Control; *p < 0.05, **p < 0.01, ***p < 0.001 vs. Cisplatin; ns = not significant. n = 6 independent biological replicates obtained from separate cell culture experiments. Scale bar = 20 μm.

Given the established correlation between oxidative stress-induced apoptosis and P38 MAPK activation (Zhang Q. et al., 2023; Debatti et al., 2017; Niemietz and Brown, 2023), we interrogated this signaling axis to determine its mechanistic contribution to ROS accumulation in HEI-OC1 cells. To this end, we performed pharmacological modulation of p38 signaling using both agonists and inhibitors to clarify its role in baicalin-mediated protection against cisplatin ototoxicity. MitoSOX™ Red staining revealed that cisplatin treatment caused a significant increase in mitochondrial ROS production compared to the control cells (p < 0.001), whereas baicalin co-treatment markedly suppressed this overproduction (p < 0.001 vs. cisplatin). Notably, co-administration of the p38 agonist anisomycin largely abolished the antioxidant effect of baicalin, resulting in ROS levels comparable to those observed in the cisplatin-only group, while inhibition of p38 signaling by SB203580 recapitulated the ROS-suppressive effect comparable to baicalin treatment alone (Figures 6a,c). TUNEL staining further confirmed that cisplatin exposure led to substantial apoptotic cell death (p < 0.001 vs. control), which was significantly alleviated by baicalin co-treatment (p < 0.001 vs. cisplatin). Similar to ROS results, anisomycin abrogated the anti-apoptotic effect of baicalin, whereas SB203580 treatment markedly reduced TUNEL-positive cells (Figures 6b,d).

At the molecular level, Western blot analysis revealed a marked increase in p-p38 protein levels following cisplatin treatment, accompanied by a reduction in p38 expression. Baicalin treatment effectively reduced p-p38 expression, an effect diminished by anisomycin but mimicked by SB203580 (Figures 6e,f). Collectively, the results demonstrate that baicalin attenuates cisplatin-induced mitochondrial ROS accumulation and apoptosis in auditory cells, at least in part, through inhibition of p38 MAPK activation.

## 4 Discussion

Cisplatin-induced ototoxicity remains a major dose-limiting adverse effect in cancer chemotherapy, leading to irreversible hearing loss primarily due to oxidative stress, mitochondrial dysfunction, and activation of multiple cell death pathways within cochlear hair cells. Recent reviews have highlighted that excessive reactive oxygen species (ROS) generation and inflammation play central roles in cochlear injury, while antioxidant and anti-inflammatory strategies represent promising protective approaches (Li et al., 2023). Natural compounds, particularly flavonoids and polyphenols, have gained increasing attention for their capacity to scavenge ROS, stabilize mitochondrial membrane potential, and modulate signaling cascades such as MAPK, NF-κB, and Nrf2 (Salah et al., 2025). Building upon these findings, the present study demonstrates that baicalin, a bioactive flavonoid derived from Scutellaria baicalensis, serves as a potent therapeutic agent against cisplatin-triggered ototoxicity. Through *in vitro* experiments using HEI-OC1 cells and cochlear explants, as well as *in vivo* studies in mice, we demonstrated that baicalin protects cochlear hair cells from cisplatin-induced damage. Furthermore, mechanistic investigations in HEI-OC1 cells revealed that this protective effect is closely associated with modulation of the ROS/p38 MAPK pathway, leading to reduced oxidative stress, restored mitochondrial function, and suppressed apoptosis. These results not only corroborate previous studies on baicalin’s protective roles in oxidative damage models (Song et al., 2021; Yuan et al., 2023), but also provide novel insights into its molecular targets and translational potential in chemotherapy-related hearing loss.

The *in vivo* experiments in our study underscore baicalin’s protection on cisplatin-induced hearing loss. In cisplatin-treated mice, baicalin co-administration significantly reduced ABR and DPOAE threshold shifts (Figure 1), indicating preserved auditory function. Meanwhile, a reduced loss of hair cells, particularly outer hair cells, was observed in the baicalin-treated group. These results are consistent with previous investigation of cisplatin-induced hearing loss, which predominantly affects outer hair cells (OHCs) in the basal cochlear turn (Campbell et al., 2007). The preferential vulnerability of outer hair cells may be attributed to their high metabolic demand and limited antioxidant defense capacity, rendering them particularly sensitive to oxidative stress (Maniaci et al., 2024). By mitigating OHC loss, baicalin may help preserve high-frequency hearing regions, a factor that directly impacts the quality of life of patients receiving cisplatin chemotherapy. To our knowledge, this study provides the first *in vivo* evidence supporting the otoprotective role of baicalin against cisplatin-induced hearing loss. One limitation of the present study is that only a single dose of baicalin was evaluated *in vivo*. Although the selected dose (30 mg/kg) was sufficient to confer significant functional and structural protection against cisplatin-induced ototoxicity, dose–response relationships and the optimal protective dose range remain to be determined. Future studies incorporating multiple dosing regimens will be necessary to define the minimal effective dose and therapeutic window of baicalin for potential clinical translation.

Our experimental data indicate that baicalin exerts robust protective effects against cisplatin-induced cytotoxicity in both HEI-OC1 cells and cochlear hair cells. This protection appears to be primarily mediated through the suppression of apoptosis, as reflected by decreased Bax and cleaved Caspase-3 expression alongside increased Bcl-2 levels, suggesting a restoration of mitochondrial apoptotic balance. Given that apoptosis is a major contributor to cochlear hair cell loss following cisplatin exposure, the anti-apoptotic effect of baicalin represents a crucial mechanism for preserving auditory function. Similar cytoprotective effects have been observed in other organ systems, including neuronal and renal models, indicating that the ability of baicalin to inhibit apoptosis is a conserved cellular response to toxic injury (Zhang and Yu, 2019; Wang et al., 2023; Liu et al., 2022). Moreover, this finding underscores the tight interplay between oxidative stress and apoptosis, as oxidative injury often precedes and amplifies apoptotic cascades. By attenuating apoptotic responses, baicalin may therefore disrupt this feed-forward cycle of oxidative and mitochondrial injury, maintaining cellular viability under toxic stress. Together, these observations suggest that baicalin’s cytoprotective effects are closely associated with its ability to modulate mitochondrial homeostasis, highlighting a conserved mechanism across different cell types. Notably, our results extend this mechanistic understanding to cochlear hair cells, providing further evidence that targeting mitochondrial apoptotic regulation is a key aspect of baicalin’s protective actions in the context of cisplatin-induced ototoxicity.

Following these observations, we further investigated how baicalin modulates mitochondrial dysfunction, a central contributor to cisplatin ototoxicity characterized by excessive ROS production and ΔΨm collapse (Fujimoto and Yamasoba, 2019). Excessive ROS has been recognized as a primary trigger of mitochondrial injury, leading to lipid peroxidation, protein oxidation, and subsequent collapse of the mitochondrial membrane potential (ΔΨm) (Fujimoto and Yamasoba, 2019; Tan and Vlajkovic, 2023). Our study demonstrates that baicalin effectively mitigates these pathological changes. Using Mito-SOX Red staining, we observed a significant reduction in mitochondrial ROS production in baicalin-treated HEI-OC1 cell lines and cochlear explants (Figure 4). Furthermore, JC-1 and TMRM assays confirmed that baicalin restores ΔΨm in cisplatin-damaged cells (Figure 5), suggesting its role in stabilizing mitochondrial integrity. Preserving ΔΨm is critical, as its depolarization represents an early hallmark of intrinsic apoptotic signaling and facilitates cytochrome c release into the cytosol (Zaib et al., 2022). By mitigating ROS overproduction and maintaining ΔΨm, Baicalin appears to interrupt this vicious cycle, stabilizing mitochondrial function and preventing apoptosis. These protective effects may involve enhancement of endogenous antioxidant defenses, potentially via activation of Nrf2/HO-1 signaling, as reported in other cellular models (Wang et al., 2021; Ma et al., 2021). However, this pathway was not directly investigated in the present study, and therefore its involvement in baicalin-mediated otoprotection remains speculative. Future studies incorporating molecular analyses of Nrf2/HO-1 signaling in cochlear tissues will be necessary to clarify this possibility.

In addition to its effects on oxidative stress and mitochondrial integrity, our data further indicate that baicalin modulates the ROS/p38 MAPK signaling pathway—a well-established mediator of cell apoptosis (Kumar et al., 2021; Heo et al., 2018). Elevated ROS levels activate p38 MAPK, which in turn amplifies apoptotic cascades and exacerbates cochlear hair cell degeneration. Consistent with this, our experiments showed that baicalin effectively suppressed cisplatin-induced phosphorylation of p38, while pharmacological manipulation with anisomycin (p38 agonist) and SB203580 (p38 inhibitor) confirmed that baicalin’s protective effects are at least partly mediated through inhibition of p38 MAPK activation. This mechanistic link is supported by studies in renal and hepatic models, where p38 MAPK inhibition attenuates cisplatin-induced organ damage (Wu et al., 2023; Bekhit et al., 2023). Although p38 MAPK activation has been implicated in stress-induced apoptosis, its precise role in cisplatin ototoxicity has not been fully elucidated. Here, we demonstrate that baicalin attenuates cisplatin-induced cytotoxicity in auditory cells *in vitro* by disrupting the ROS/p38 MAPK axis, providing a mechanistic explanation for its observed protective effects at the cellular level. Moreover, because the ROS/p38 pathway also influences inflammatory cytokine release (e.g., TNF-α, IL-6) and DNA repair responses (Zhang Y. et al., 2023; Zhang et al., 2025), our findings raise the possibility that baicalin may exert broader anti-inflammatory and genoprotective effects *in vitro*, although these potential actions remain to be validated *in vivo*.

Apart from the p38 MAPK pathway, other stress-related signaling cascades—including JNK, PI3K/AKT, and NF-κB—also play pivotal roles in hair cell survival following ototoxic insults (Salah et al., 2025; Liu et al., 2021). Given baicalin’s broad antioxidant and anti-inflammatory properties, its protective effects may extend to these additional pathways. Interestingly, such modulation appears to be context-dependent—baicalin may suppress PI3K/Akt or MAPK activity in tumor cells to induce apoptosis, whereas in neuronal and sensory cells it tends to activate these pathways to enhance survival and mitochondrial stability (Li et al., 2021; Huang et al., 2012). This context-specific regulation underscores baicalin’s ability to fine-tune cellular signaling in response to different stress environments, which may explain its broad yet selective cytoprotective effects.

Moreover, upstream regulators such as apoptosis signal-regulating kinase 1 (ASK1), a redox-sensitive kinase that activates both p38 and JNK under oxidative stress (Meijles et al., 2020), and endogenous antioxidant enzymes such as superoxide dismutase (SOD) and catalase (CAT) (Jomova et al., 2024), may serve as critical nodes for therapeutic intervention. Future studies should therefore explore these alternative mechanisms using pharmacologic inhibitors or genetic silencing approaches to delineate whether baicalin’s effects are mediated through multi-pathway modulation. Such insights would help refine its therapeutic potential and inform rational combination strategies for preventing cisplatin-induced ototoxicity. However, the mechanistic investigations in this study were primarily conducted *in vitro*, whereas the *in vivo* experiments mainly confirmed structural and functional preservation. Thus, the molecular pathways responsible for baicalin’s otoprotective effects remain to be fully validated in animal models. Since cisplatin is primarily used for cancer treatment, it is critical to evaluate baicalin in tumor-bearing animal models to determine whether its cochlear protection interferes with cisplatin’s antitumor efficacy. Future studies integrating molecular analyses with auditory function tests in such models will be essential to confirm both the safety and translational applicability of baicalin in clinical contexts.

In conclusion, this study demonstrates that baicalin protects against cisplatin-induced cochlear hair cell damage *in vivo*, as evidenced by preserved auditory function and hair cell survival. Moreover, *in vitro* mechanistic analyses indicate that baicalin-mediated cytoprotection is associated with inhibition of the ROS/p38 MAPK–mediated apoptotic pathway. These findings strengthen the rationale for baicalin as a potent natural antioxidant and underscore its promise as a therapeutic candidate for the prevention of chemotherapy-induced hearing loss.

## Data Availability

The raw data supporting the conclusions of this article will be made available by the authors, without undue reservation.
