# Correction: Baicalin attenuates cisplatin-induced cochlear hair cell damage by modulating the ROS-p38 MAPK signaling pathway

**DOI:** 10.3389/fcell.2026.2014328

**Published:** 2026-10-29

**Authors:** Qiongmin Zhang, Yating Wang, Chunhong Zhang, Sisi Li, Qian Yin, Chunchun Zhou, Chunguang Dong, Chuan Bu

**Affiliations:** 1 Department of Otorhinolaryngology, The First Affiliated Hospital of Wenzhou Medical University, Wenzhou, China; 2 Department of Otorhinolaryngology, The Affiliated Lianyungang Hospital of Xuzhou Medical University/The First People’s Hospital of Lianyungang, Lianyungang, China; 3 Department of Otorhinolaryngology, The First Affiliated Hospital of Kangda College of Nanjing Medical University/The First People’s Hospital of Lianyungang, Lianyungang, China

**Keywords:** apoptosis, baicalin, cisplatin, cochlear hair cell, ROS/P38 MAPK signaling

There was a mistake in Figure 2 as published. During the assembly of the figures, incorrect images were inadvertently uploaded, namely the Control-mid and Cis + Bai-base images in Figure 2d. The corrected Figure 2 appears below.

**FIGURE 2:**
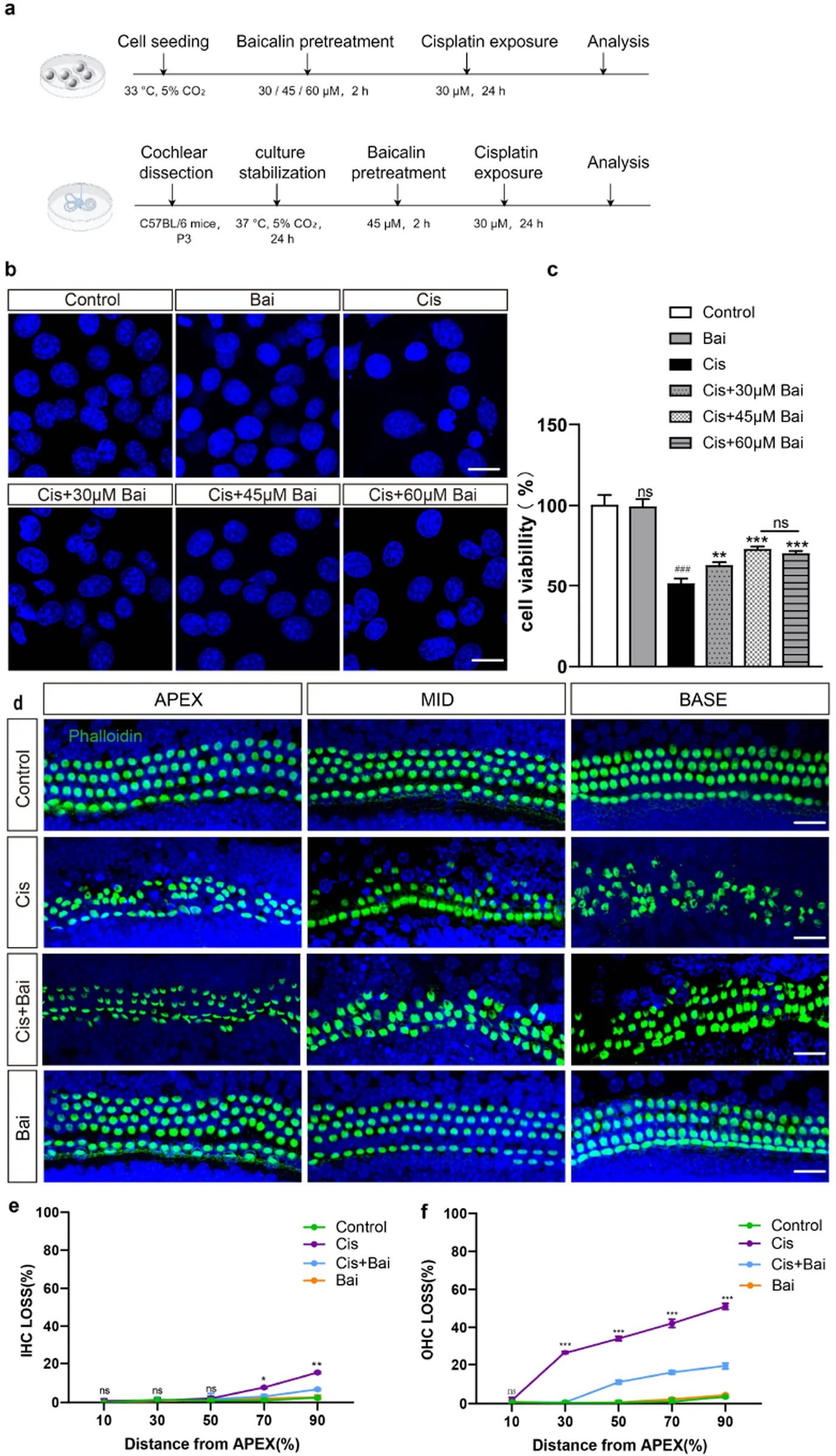
Baicalin protects against cisplatin-induced ototoxicity in HEI-OC1 cells and cochlear hair cells. **(a)** The experimental workflow for **(b–f)**. **(b)** Representative images of cell viability under different treatment conditions. Cisplatin (30 μM) treatment led to a marked decrease in cell viability relative to the untreated group. Co-treatment with various concentrations of baicalin (30, 45, 60 μM) partially restored cell viability in a dose-dependent manner. **(c)** Quantitative analysis of cell viability in different groups (control, Bai, Cis, Cis +30 μM Bai, Cis +45 μM Bai, and Cis +60 μM Bai). Data are presented as mean ± SD from three independent biological experiments, each performed in triplicate (n = 3). Statistical analysis was conducted using one-way ANOVA followed by Tukey’s *post hoc* test. ^###^p < 0.001 vs. control,**p < 0.01 vs. Cis,***p < 0.001 vs. Cis, ns = not significant. **(d)** Immunofluorescence images showing the organization of inner hair cells (IHCs) and outer hair cells (OHCs) across the cochlear apex, mid, and base regions. Phalloidin (green) staining shows the actin cytoskeleton of hair cells. Cisplatin-treated groups show HC loss, especially in the mid and base regions, while co-treatment with baicalin provided substantial protection against cisplatin-triggered hair cell injury. **(e,f)** The mean cochleogram showed HCs loss across different cochlear regions (apex to base) in control, Cis, Cis + Bai, and Bai groups. Significant HCs loss was observed in the Cis group, especially in the mid and base regions. Baicalin co-treatment reduced this loss. **p < 0.01, ***p < 0.001. For **(a,b)**, n = 6 independent biological replicates obtained from separate cell culture experiments. For **(c–e)**, n = 6 cochlear explants derived from six individual P3 mice. Scale bar = μm.

There was a mistake in Figure 3 as published. During the assembly of the figures, incorrect images were inadvertently uploaded, namely the Cis + Bai group in Figure 3a. The corrected Figure 3 appears below.

**FIGURE 3:**
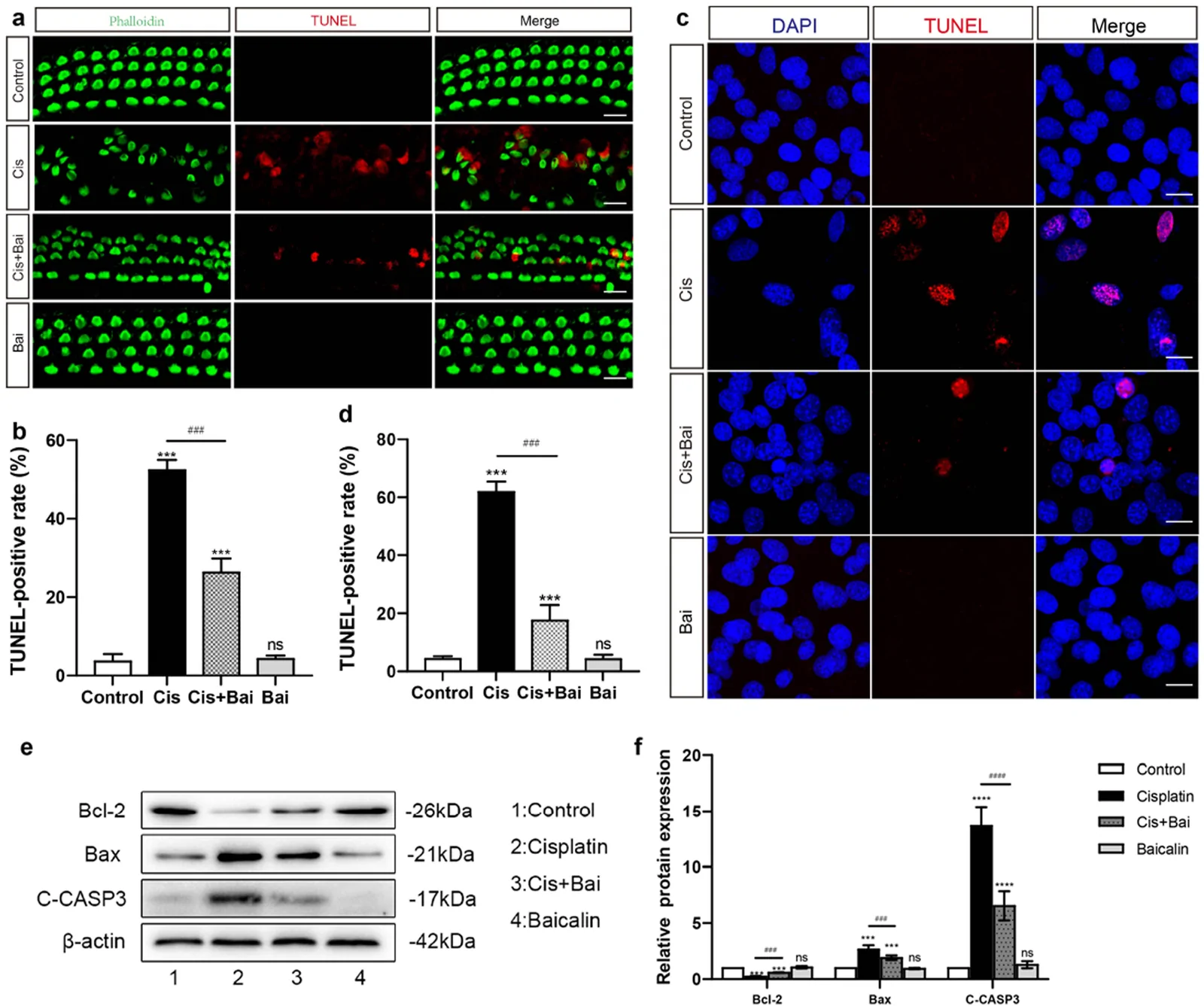
Baicalin protect cells from apoptosis in HEI-OC1 cell line and murine cochlear HCs after cisplatin injury *in vitro*. **(a,b)** Immunofluorescence images of cochlear hair cells showing p halloidin (green) and TUNEL (red) staining in the base regions. Cisplatin treatment resulted in increased TUNEL-positive apoptotic cells relative to the control group, while baicalin co-treatment notably decreased the quantity of apoptotic cells. **(c,d)** DAPI (blue) and TUNEL (red) staining of nuclei in HEI-OC1 cells, showing increased apoptotic cell death in the Cis group, while baicalin co-treatment significantly decreased TUNEL-positive cells. **(e,f)** Western blot analysis demonstrating quantitative alterations of Bcl-2, Bax, and cleaved C-CASP3 in different treatment groups. Cisplatin treatment decreased Bcl-2 level and elevated Bax and C-CASP3 levels. Baicalin co-treatment restored Bcl-2 levels and reduced Bax and C-CASP3 expression. Data are presented as mean ± SD (n = 3), ***p < 0.001,****p < 0.0001,ns vs. the control group, ^###^P < 0.001,^####^P < 0.0001, ns = not significant. For **(a,b)**, n = 6 cochlear explants derived from six individual P3 mice. For **(c,d)**, n = 6 independent biological replicates obtained from separate cell culture experiments. Scale bar = 20 μm.

There was a mistake in Figure 5 as published. During the assembly of the figures, incorrect images were inadvertently uploaded, namely the Control group in Figure 5a. The corrected Figure 5 appears below.

**FIGURE 5:**
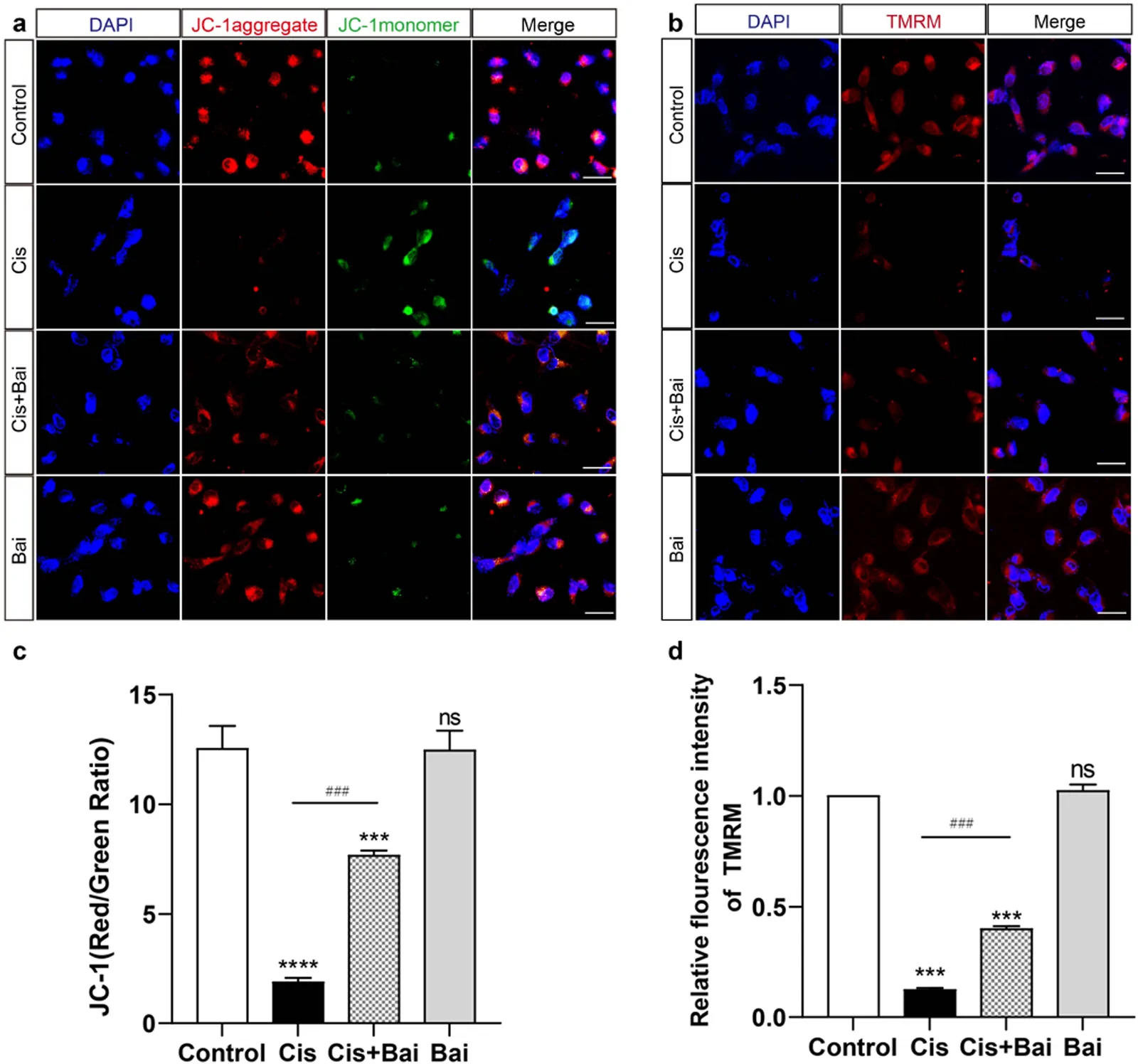
Baicalin preserves mitochondrial membrane potential in cisplatin-damaged HEI-OC1 cells. **(a–c)** JC-1 fluorescence micrographs and corresponding ratiometric analysis (aggregate/monomer fluorescence intensity) demonstrating Baicalin-mediated preservation of mitochondrial transmembrane potential in cisplatin-challenged HEI-OC1 auditory cells. The JC-1 fluorescence (red/green ratio) in Cis group significantly decreased, while co-treatment with Bai raised the JC-1 fluorescence (red/green ratio) in the Cis + Bai group. **(b–d)** Representative images of TMRM staining showed different mitochondria changes in the same treatment groups as a. The relative fluorescence intensity of TMRM was markedly decreased in the Cis group, reflecting a decrease in mitochondrial membrane potential, while the Cis + Bai group showed restored mitochondrial membrane potential to Cis alone. Fluorescence intensity was normalized to the control group (control = 1.0). ***p < 0.001, ****p < 0.0001,ns vs. the control group,^###^P < 0.001, ns = not significant. n = 6 independent biological replicates obtained from separate cell culture experiments. Scale bar = 20 μm.

The original version of this article has been updated.

